# Multitargeted Virtual Screening and Molecular Simulation of Natural Product-like Compounds against GSK3β, NMDA-Receptor, and BACE-1 for the Management of Alzheimer’s Disease

**DOI:** 10.3390/ph16040622

**Published:** 2023-04-20

**Authors:** Danish Iqbal, Md Tabish Rehman, Mohamed F. Alajmi, Mohammed Alsaweed, Qazi Mohammad Sajid Jamal, Sharifa M. Alasiry, Awatif B. Albaker, Munerah Hamed, Mehnaz Kamal, Hind Muteb Albadrani

**Affiliations:** 1 Department of Health Information Management, College of Applied Medical Sciences, Buraydah Private Colleges, Buraydah 51418, Saudi Arabia; 2 Department of Pharmacognosy, College of Pharmacy, King Saud University, Riyadh 11451, Saudi Arabia; mrehman@ksu.edu.sa (M.T.R.); malajmii@ksu.edu.sa (M.F.A.); 3 Department of Medical Laboratory Sciences, College of Applied Medical Sciences, Majmaah University, Majmaah 11952, Saudi Arabia; m.alsaweed@mu.edu.sa; 4 Department of Health Informatics, College of Public Health and Health Informatics, Qassim University, Al Bukayriyah 52741, Saudi Arabia; m.quazi@qu.edu.sa; 5 Critical Care Nursing, Department of Nursing, College of Applied Medical Sciences, Majmaah University, Al-Majmaah 15341, Saudi Arabia; s.alasiry@mu.edu.sa; 6 Department of Pharmacology and Toxicology, College of Pharmacy, King Saud University, Riyadh 11451, Saudi Arabia; abaker@ksu.edu.sa; 7 Department of Pathology, Faculty of Medicine, Umm Al-Qura University, Makkah 21955, Saudi Arabia; mhhamed@uqu.edu.sa; 8 Department of Pharmaceutical Chemistry, College of Pharmacy, Prince Sattam Bin Abdulaziz University, Al-Kharj 11942, Saudi Arabia; mailtomehnaz@gmail.com

**Keywords:** Alzheimer’s disease, natural product, enzyme inhibitors, GSK3β, NMDA-receptor, BACE-1, virtual screening

## Abstract

The complexity of Alzheimer’s disease (AD) and several side effects of currently available medication inclined us to search for a novel natural cure by targeting multiple key regulatory proteins. We initially virtually screened the natural product-like compounds against GSK3β, NMDA receptor, and BACE-1 and thereafter validated the best hit through molecular dynamics simulation (MDS). The results demonstrated that out of 2029 compounds, only 51 compounds exhibited better binding interactions than native ligands, with all three protein targets (NMDA, GSK3β, and BACE) considered multitarget inhibitors. Among them, F1094-0201 is the most potent inhibitor against multiple targets with binding energy −11.7, −10.6, and −12 kcal/mol, respectively. ADME-T analysis results showed that F1094-0201 was found to be suitable for CNS drug-likeness in addition to their other drug-likeness properties. The MDS results of RMSD, RMSF, Rg, SASA, SSE and residue interactions indicated the formation of a strong and stable association in the complex of ligands (F1094-0201) and proteins. These findings confirm the F1094-0201’s ability to remain inside target proteins’ binding pockets while forming a stable complex of protein-ligand. The free energies (MM/GBSA) of BACE-F1094-0201, GSK3β-F1094-0201, and NMDA-F1094-0201 complex formation were −73.78 ± 4.31 kcal mol^−1^, −72.77 ± 3.43 kcal mol^−1^, and −52.51 ± 2.85 kcal mol^−1^, respectively. Amongst the target proteins, F1094-0201 have a more stable association with BACE, followed by NMDA and GSK3β. These attributes of F1094-0201 indicate it as a possible option for the management of pathophysiological pathways associated with AD.

## 1. Introduction

Alzheimer’s disease is a well-known neurological disorder whose pathophysiology is quite complex in nature, which is increasing in nearly every country. AD and other dementia types are expected to rise to 78 million cases which could increase the global societal cost to 2.8 trillion (US dollars) by 2030. Patients with AD have symptoms like declining cognitive skills and memory loss, exhibited mainly in people of old age [1,2]. Among AD patients, it was observed that hyperactivation of several proteins, including GSK3β, NMDA-receptor, and BACE-1, could cause deposition of amyloid plaques and/or formation of neurofibrillary tangles, which can lead to deterioration of nerve cells and negatively effects neurotransmission [3]. β-secretase (BACE) is the critical protein for the cleavage of amyloid precursor protein into amyloidogenic peptides (Aβ_42_), which aggregates to form amyloid plaques. Furthermore, the deposition of amyloid plaques and oxidative stress can result in the hyperactivation of GSK3β, which increases the phosphorylation of tau proteins and potentiate the formation of neurofibrillary tangles [4]. Moreover, the hyperactivation of NMDA-receptor (NMDA) in combination with neurofibrillary tangles and amyloid plaques raises the release of glutamate, which could lead to excitotoxicity and neuronal death [5,6]. 

Previous reports established that by inhibiting the essential proteins, such as cholinesterase, monoamine oxidases, ROCK-2, Calcium homeostasis modulator-1 (CALMH1), Glycogen synthase kinase-3 beta (GSK3β), N-methyl-D-aspartate (NMDA) receptor, and beta-site APP cleaving enzyme 1 (BACE-1 or BACE) can lessen the memory loss and neuronal death [3,7,8,9,10,11]. FDA-approved medications for the treatment of AD are mostly antagonists of crucial proteins involved in the pathogenesis of the disease. However, due to several side effects in long-term use, like vomiting, nausea, loss of appetite, constipation, headache, confusion, and dizziness, there is a continuous demand for novel compounds having lower side effects and are cost-effective [12]. FDA has approved a non-competitive inhibitor (memantine) of NMDA for the treatment of mild AD. Despite several clinical trials searching for a potential cure for AD [13], there are still no disease-modifying AD drugs [14]. 

Due to the complexity of Alzheimer’s disease, there are several proteins involved in its pathogenesis, multitarget directed ligands (MTDLs) as potential therapeutic agents for the different pathological cascades of AD in comparison to single/multitarget approaches for a single pathway of AD progression are considered to be a better cure for the disease [8,15,16,17,18,19].

Natural products and their derivatives were found to have better therapeutic potentials against several metabolic and infectious disorders such as diabetes, oxidative stress, hyperlipidemia, ulcer, neurodegenerative disorders, cancer and microbial infections [20,21,22,23,24,25,26,27,28,29,30,31,32,33,34,35,36,37,38]. Earlier reports explored the therapeutic roles and mechanism of action of polyphenols against age-related neurological disorders [39,40]. The plants *Grifola frondosa* and *Hericium erinaceus* are excellent sources of β-glucans and have better anti-aging properties, whereas polyphenols of extra virgin olive oil (oleuropein aglycone and hydroxytyrosol) synergistically modulate autophagy against neurodegeneration due to their strong antioxidant and anti-inflammatory properties [40,41,42]. Nature provides an abundant resource of bioactive metabolites, which could be safe and economical to produce novel inhibitors of critical regulatory enzymes for the management of metabolic disorders, including neurological diseases [29,43,44]. The library of natural product-like compounds is a database of purchasable compounds, including the derivatives of natural compounds as well as pure natural compounds [8,45,46].

Therefore, this study investigated the multitargeted therapeutic potentials of a library of natural product-like compounds (NPLC) against GSK3β, NMDA-receptor, and BACE-1 through molecular docking and ADMET tools. Moreover, we validated the best hit through molecular dynamics simulation (MDS). To the precise of our knowledge, this library of compounds has never been explored for targeting the GSK3β, NMDA-receptor, and BACE-1 for managing AD.

## 2. Results and Discussion

### 2.1. Virtual Screening of Natural Product-like Compounds 

Computational screening of a vast number of small organic molecules for their inhibitory potential against the target proteins is widely recognized and accepted, which can significantly reduce the time, cost and efforts of wet lab high-throughput screening [47,48,49,50]. In this study, we applied virtual screening through molecular docking and validation of best hits through molecular dynamics simulation (MDS) to identify the novel inhibitor for multiple targets (NMDA, GSK3β, and BACE) to manage neurological disorders. Primarily, the molecular docking protocol was validated by redocking the native ligands and found that it bounds to almost similar residues. RMSD value (≤2) between docked and native ligands was in an acceptable limit. Then, the natural product-like compounds (NPLC) library and reference inhibitors/substrate (Native ligand of GSK3β: Adenosine-5′-Diphosphate, Native ligand of NMDA receptor: 5,7-Dichlorokynurenic acid, and Native ligand of BACE: non-peptidic inhibitor) were individually docked on the active site of target proteins NMDA, GSK3β, and BACE, respectively. The results demonstrated that out of 2029 compounds total of 288 compounds have binding energy scores between −10 and −12.3 Kcal/mol for NMDA, 135 compounds exhibited binding energy scores between −9 and −11.2 Kcal/mol for GSK3β, and 213 compounds showed binding energy scores between −10 and −12 Kcal/mol for BACE. Of these best active 288, 135, and 213 compounds against NMDA, GSK3β, and BACE, respectively, only 51 compounds exhibited binding interactions with all three targets and were considered multitarget inhibitors. The top 10 best hits of compounds against all three targets were chosen (Table 1) for further analysis. 

### 2.2. Drug-Likeness, Pharmacokinetics, and Physicochemical Properties

This study used the SwissADME tool to analyze the physicochemical, pharmacokinetics, and drug-likeness properties of the 10 best hits (multiple targets inhibitors; Table 2). The significance of adhering to these parameters was well established. It has been said that most medications failed throughout the drug development process because they were ineffective at adhering to these parameters [51,52]. The physiological properties of the majority of orally active drugs, such as molecular weight (MW), hydrogen bond donors (HBD), hydrogen bond acceptors (HBA), and XlogP, were found to be in a specified range (MW: 160–500 g/mol, HBD: 5, HBA: 10, and XlogP: −1–6). Poor oral bioavailability is represented by chemical structures with more than 10 rotatable bonds [53]. In addition, the molar refractivity (MR) range was regarded as between 40 and 130 for more excellent intestine absorption [54]. Therefore, according to Lipinski et al. (1997), the compounds must adhere to the acceptable range for at least three physiochemical attributes out of five to be considered drug-like [52].

**Figure 1:**
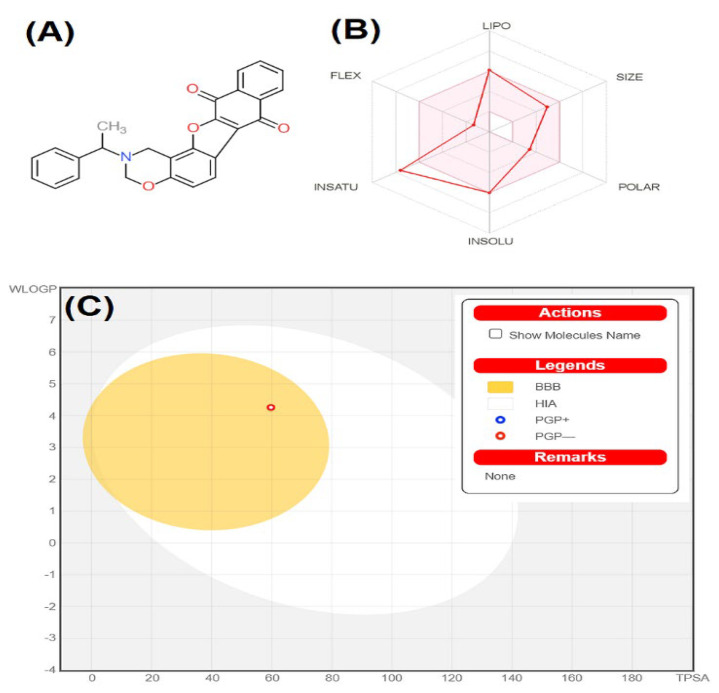
(**A**) Structure of F1094-0201 (2−(1-phenylethyl)−2,3-dihydro−1H-naphtho[2′,3′:2,3]benzofuro[7,6-e][1,3]oxazine−7,12-dione); (**B**) acceptable range (pink-colored region) for pharmacokinetics properties of F1094-0201; (**C**) description about BOILED-Egg image for F1094-0201 to predict gastrointestinal absorption (HIA) and brain penetration (BBB).

Our results (Table 2) illustrated that out of the 10 best hits, only five (F1094-0201, F1217-0041, F1094-0205, F1094-0196, and F1094-0198) compounds could penetrate the blood-brain barrier (BBB) which is the most important property for any central nervous system medication [55]. Furthermore, all the compounds except F3161-0307 showed high GI absorption, whereas five (F0870-0001, F1217-0041, F1094-0205, F1094-0196, and F1094-0206) compounds were found to be Pgp-substrate. Those compounds exhibited as good Pgp-substrate are unsuitable for CNS drug candidates [56]. These results illustrate that all 10 best hits except one compound (F3161-0307) followed the Lipinski rule of five, having an acceptable range of MW, HBD, HBA, XlogP, and MR. Therefore, after the analysis of these results, only two compounds (F1094-0201 and F1094-0198) were found to be suitable for CNS drug-likeness in which BOILED-Egg image for F1094-0201 to predict gastrointestinal absorption (HIA) and brain penetration (BBB) has been shown due to its higher inhibitory potential against multiple targets (Figure 1). Our results also illustrated that all the best hits follow Veber’s rule as they have fewer than 10 rotatable bonds, and the TPSA is under the acceptable limit for drug-likeness, which is less than 140 Å^2^ which showed that our best hits have good oral bioavailability and better penetration possibility [53].

### 2.3. Molecular Docking Study

**Figure 2:**
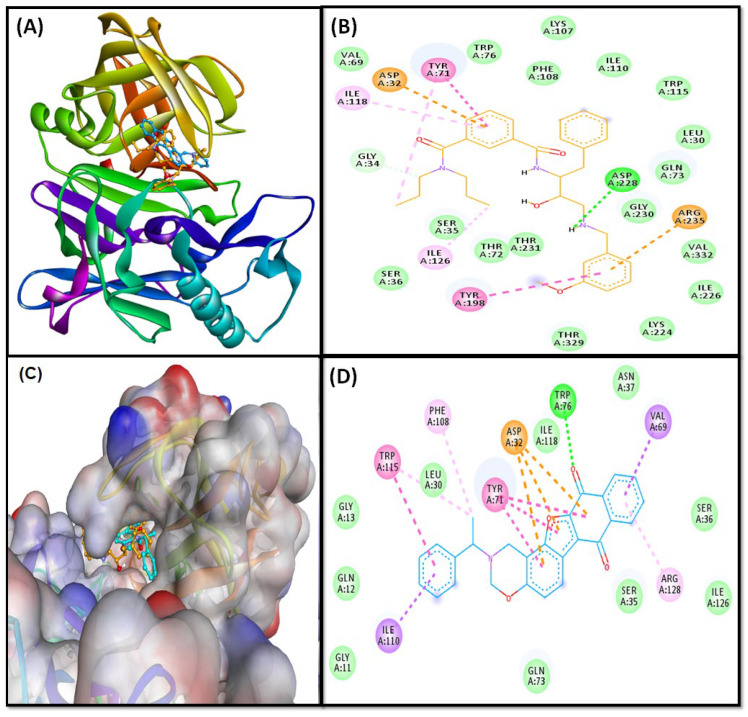
(**A**) Ligands bound to the catalytic site of the target protein (BACE); (**B**) molecular interactions between reference ligand (non-peptidic inhibitor) and target protein (BACE); (**C**) zoom-in image of ligands-BACE complex; (**D**) molecular interactions between F1094-0201 and target protein (BACE).

**Figure 3:**
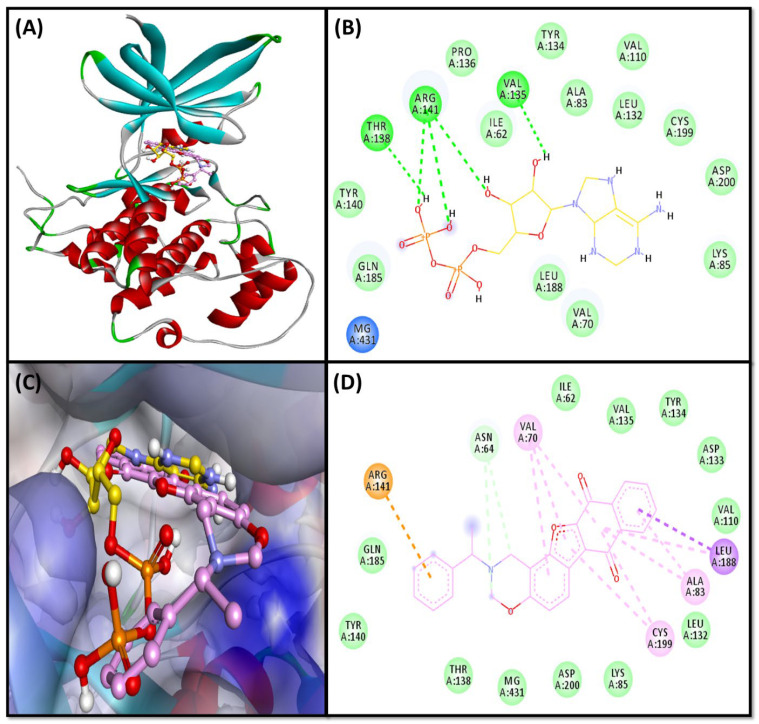
(**A**) Ligands bound to the catalytic site of the target protein (GSK3β); (**B**) Molecular interactions between reference ligand (Adenosine-5′-Diphosphate) and target protein (GSK3β); (**C**) Zoom-in image of ligands- GSK3β complex; (**D**) Molecular interactions between F1094-0201 and target (GSK3β).

**Figure 4:**
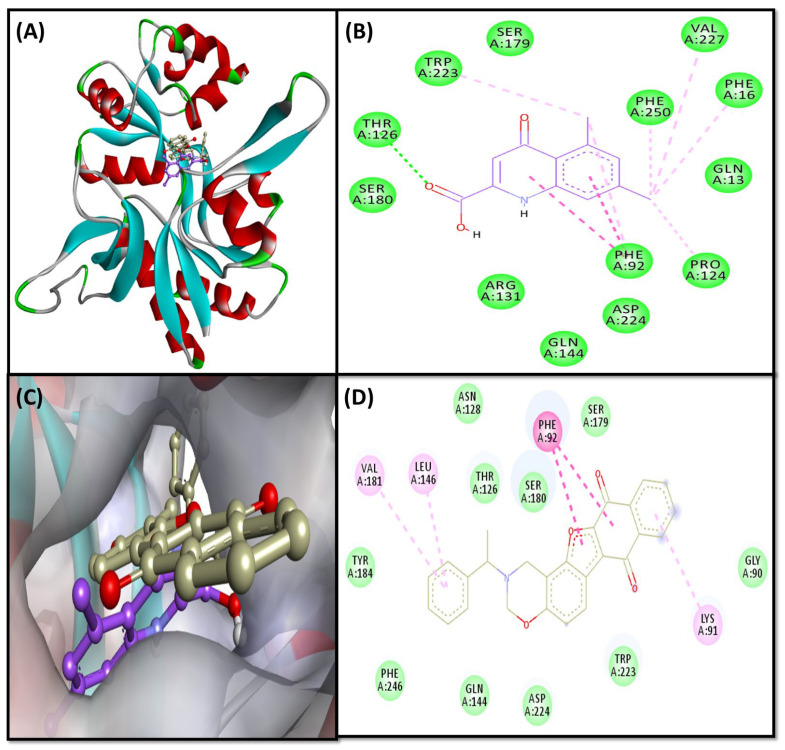
(**A**) Ligands bound to the catalytic site of the target protein (NMDA); (**B**) Molecular interactions between reference inhibitor (5,7-Dichlorokynurenic acid) and target protein (NMDA); (**C**) Zoom-in image of the ligand-NMDA complex; (**D**) Molecular interactions between F1094-0201 and target (NMDA).

The virtual screening of two thousand twenty-nine natural product-like compounds and blood-brain barrier penetration capacity of the top 10 best hits illustrate that the F1094-0201 is the most effective inhibitor against multiple targets (NMDA, GSK3β, and BACE) with binding energy −11.7, −10.6, and −12 kcal/mol, respectively. Further analysis of the ligand-protein complex revealed the interacting amino acid residues and the type of interactions involved in stabilizing the complexes. The interactions of F1094-0201 with the active site residues of NMDA, GSK3β, and BACE are shown in Figure 2, Figure 3 and Figure 4, respectively.

#### 2.3.1. BACE Complex

Our results revealed that F1094-0201 and reference inhibitor (native ligand: non-peptidic inhibitor) bound to the catalytic site of the target protein (BACE; Figure 2A–C). the non-peptidic inhibitor and BACE complex are stabilized by one conventional hydrogen bond between LIG: H—ASP228:OD2, one carbon-hydrogen bond between LIG: C—GLY34:O, two electrostatic interactions with ASP32, and ARG235 residues, two Pi-Pi stacked interactions with TYR71 and TYR198 residues, one alkyl interaction with ILE126, and two Pi-alkyl interactions with TYR71 and ILE118. Moreover, this complex also showed van der Waals interaction with several residues (LEU30, SER35, SER36, VAL69, THR72, GLN73, TRP76, LYS107, PHE108, ILE110, TRP115, LYS224, ILE226, THR231, THR329, and VAL332; Figure 2B). In contrast, F1094-0201 and the BACE complex were stabilized by one Conventional H-Bond between TRP76:HE1—LIG:O, three Pi-anion (electrostatic) interactions with ASP32 residue, and nine hydrophobic interactions in which two Pi-sigma between VAL69:CG1—LIG, and ILE110:CD1—LIG, four Pi-Pi Stacked interactions with TYR71, and TRP115 residues, and three Pi-alkyl interactions with PHE108, TRP115, and ARG128 residues. Furthermore, several van der Waals interactions were also observed with GLY11, GLN12, GLY13, LEU30, SER35, SER36, ASN37, GLN73, ILE118, and ILE126 residues (Figure 2D). Interestingly, the amino acid residues of BACE commonly interacted with F1094-0201, and non-peptidic inhibitors are LEU30, SER35, VAL69, TYR71, GLN73, TRP76, PHE108, ILE110, TRP115, and ILE118. Furthermore, we found that F1094-0201 bound with 10.6-fold higher affinity with BACE than a non-peptidic inhibitor.

Elevated beta-secretase (BACE) activity may have harmful effects on CNS due to its involvement in the production of Aβ_42_, known for forming AB plaque by protein aggregation. Reduction of Aβ_42_ toxicity in SH-SY5Y human neuroblastoma cells was achieved by polyphenols which modulate autophagy against neurodegeneration [40]. Moreover, some neurological disorders are accompanied by α-synuclein (α-syn) misfolding, and reports suggested that fungal extracts known to reduce α-syn toxicity through diverse processes, such as the reduction of protein aggregation, a decrease of the ROS level, and α-syn membrane delocalization supporting their anti-aggregation properties. Hence the reduction of protein aggregates can be a better approach to managing neurological disorders [42]. Therefore, BACE is considered a prime target for delaying amyloid pathology and managing AD [3,57,58]. We used BACE enzyme co-crystallized with hydroxyethyl amine inhibitor at 2.55 Å resolution [59]. The two aspartate (ASP32 and ASP228) residues are involved in the catalytic process of the enzyme [60,61]. Our results showed that a critical catalytic residue ASP32 interacted with the F1094-0201. Our results correspond with the previous report, stating that apart from ASP 32 and ASP228, the BACE inhibitors also interacted with GLY34, TYR71, LYS107, and PHE108 [62].

#### 2.3.2. GSK3β-Complex

Natural product-like compound (F1094-0201) and reference ligand (Substrate: Adenosine-5′-Diphosphate) interaction with GSK3β protein is shown in Figure 3. We observed that both the ligands bound to the active site residues in the same catalytic gorge (Figure 3A–C). The Adenosine-5′-Diphosphate and GSK3β complexes were stabilized through five conventional hydrogen bonds with ARG141, VAL135, and THR138 residues. Meanwhile, van der Waals’ interactions were also observed with several residues (ILE62, VAL70, ALA83, LYS85, VAL110, LEU132, TYR134, PRO136, TYR140, GLN185, LEU188, CYS199, and ASP200; Figure 3B). Moreover, F1094-0201 and GSK3β complex was stabilized by two carbon hydrogen bonds with ASN64 residue, one electrostatic (Pi-cation) interaction with ARG141, one Pi-sigma hydrophobic interactions with LEU188, five Pi-alkyl hydrophobic interactions with ALA83, LEU188, CYS199 residues, and several van der waals interactions with ILE62, LYS85, VAL110, LEU132, ASP133, TYR134, VAL135, THR138, TYR140, GLN185, and ASP200 residues (Figure 3D). Interestingly, the amino acid residues of BACE commonly interacted with F1094-0201 and Adenosine-5′-Diphosphate are ILE62, ALA83, LYS85, VAL110, LEU132, TYR134, VAL135, THR138, TYR140, ARG141, GLN185, LEU188, CYS199, and ASP200. We found that F1094-0201 bound with 133.6-fold higher affinity with GSK3β compared to Adenosine-5′-Diphosphate.

Previous in-vitro studies suggested that the production of toxic Aβ peptide can also be regulated by GSK3β via affecting presenilin-1 function. Studies conducted in-vitro and in transgenic AD animal models showed that Aβ stimulates GSK3β signaling; further, the brains of AD patients showed a comparable rise in GSK3β activity. Hyperactivation of GSK3β increases the phosphorylation of tau proteins and potentiates the formation of neurofibrillary tangles. However, through the implementation of an NF-kB signaling-mediated approach, GSK3β inhibition decreases BACE1-mediated breakage of APP. This finding consequently implies that inhibiting GSK3β lessens the disease associated with Aβ pathology [63].

GSK3β protein has two active sites: ATP-binding and substrate-binding sites, where key residues (VAL135 and ASP133) are available at the ATP-binding site, known as the activation loop. Whereas LYS85 and GLU97 also have a prominent role in the catalytic process [64]. The previous report suggests that ARG141 is one of the essential residues for specific ATP/ADP recognition by TPK I/GSK3 beta, and several other residues are of key importance in ATP-binding sites, including ILE62, VAL70, ALA83, LYS85, VAL110, LEU132, GLN185, LEU188, and ASP200 [65]. Our results demonstrate that both the reference ligand (Adenosine-5′-Diphosphate) and F1094-0201 compound commonly interacted with most of the key residues (ILE62, ALA83, LYS85, VAL110, LEU132, GLN185, LEU188, and ASP200). Moreover, F1094-0201 was also found to interact with ASP133. Our results correspond with previously published reports [64,66].

#### 2.3.3. NMDA-Complex

The binding interaction pattern of F1094-0201 and reference ligand (inhibitor: 5,7-Dichlorokynurenic acid) revealed that both the ligands occupied the active site gorge residues (Figure 4A–C). The reference inhibitor bound to the similar active site residues (GLN 13, PHE16, PHE92, PRO124, THR126, ARG131, GLN144, SER179, SER180, ASP224, VAL227, and PHE250) in NMDA via hydrogen bond, hydrophobic and van der Waals interactions (Figure 4B). The molecular interactions analysis exhibited that F1094-0201 and NMDA complex was stabilized by several intermolecular interactions, where one conventional hydrogen bond of distance 2.48 A was observed (THR126:HG1—LIG:O), eight hydrophobic interactions between catalytic residues (PHE92, PRO124, PHE16, TRP223, PHE250) of protein and ligand via two Pi-Pi Stacked (PHE92—LIG), one Alkyl (LIG: Cl—PRO124), and five Pi-Alkyl (PHE16—LIG: Cl, PHE92—LIG: Cl, TRP223—LIG: Cl, and PHE250—LIG: Cl). Moreover, 10 van der Waals interactions between ligand and protein residues (GLY90, THR126, ASN128, SER179, SER180, TYR184, TRP223, ASP224, PHE246) were also involved in stabilizing the complex (Figure 4D). Interestingly, the amino acid residues of NMDA commonly interacted with F1094-0201 and 5,7-Dichlorokynurenic Acid are PHE16, PHE92, PRO124, THR126, SER179, SER180, ASP224, and PHE250. We found that F1094-0201 bound with a 1988.6-fold higher affinity with NMDA than 5,7-Dichlorokynurenic acid.

Neuronal survival is reliant on synaptic NMDA receptor signaling. The overflow of glutamate produced by astrocytes or presynaptic terminals plays a crucial role in antagonizing the synaptic pro-survival signaling pathway and tipping the scales in favor of excitotoxicity and eventual neurodegeneration. Memantine, an FDA-approved NMDA receptor antagonist, has been shown to have positive therapeutic benefits in moderate-to-severe AD patients. It may do this by reducing extra-synaptic NMDA receptor signaling. Therefore it is beneficial to target NMDA receptors for the management of AD [67].

NMDA receptors require both glycine and glutamate for activation, with NR1 and NR2 forming glycine and glutamate sites, respectively. Here we used the high-resolution (1.90 Å) co-crystal structures of the NR1 S1S2 ligand-binding core with the antagonist 5,7-dichloro kynurenic acid (DCKA). The NR1 site has been considered for therapeutic potential [68,69]. Ugale and Bari reported that amino acid residues Arg131, Pro124, and Thr126 are essential for inhibiting the Gly/NMDA receptor [70,71]. These interactions were also noticed by Devid et al. [72] with some additional interactions (GLN13, ASP224, and Trp223 residues). Our results are in correspondence with these previous reports.

### 2.4. Analysis of Molecular Dynamics Simulation

#### 2.4.1. Root Mean Square Deviation (RMSD)

**Figure 5:**
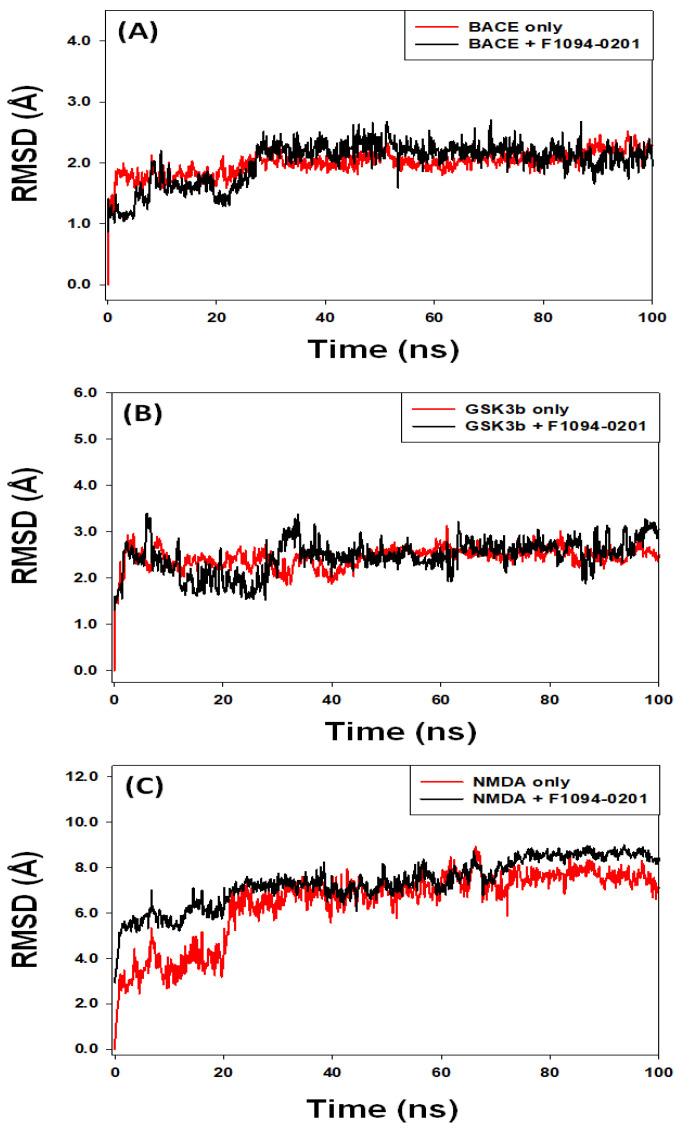
Variation in the root mean square deviation (RMSD) of (**A**) BACE, (**B**) GSK3β, and (**C**) NMDA, in the absence or presence of F1094-0201 as a function of simulation time.

A protein-ligand complex’s RMSD is often calculated to assess the stability and dynamic properties of the complex. The RMSD is determined by deviating from the protein-ligand starting pose’s structure as a function of simulation duration [73]. In this experiment, we measured the RMSD of three target proteins (BACE, GSK3, and NMDA) in the presence of the chemical F1094-0201 throughout a simulation time of 100 ns (Figure 5). The RMSD of BACE increased during the first few seconds and remained consistent throughout the simulation. However, the RMSD of BACE in the presence of F1094-0201 fluctuated during 0–30 ns and then attained an equilibrium for 30–100 ns time. The average RMSD values of BACE in the absence and presence of F1094-0201 during 30–100 ns were 2.04 ± 0.36 Å and 2.18 ± 0.71 Å, respectively (Figure 5A). Similarly, the RMSD of GSK3β in the absence and presence of F1094-0201 fluctuated insignificantly during 0–35 ns and remained consistent within 2.0–3.4 Å throughout the simulation time. The average RMSD of GSK3β alone or the GSK3β-F1094-0201 complex during 35–100 ns were estimated to be 2.54 ± 0.61 Å and 2.78 ± 0.57 Å, respectively (Figure 5B). Likewise, the RMSD of NMDA in the absence and presence of F1094-0201 fluctuated during 0–20 ns and then equilibrated for the rest of the simulation time. The average RMSD of NMDA and the NMDA-F1094-0201 complex during 20–100 ns was 6.73 ± 0.76 Å and 7.85 ± 0.59 Å, respectively (Figure 5C).

The MDS results indicated the establishment of a stable association between proteins and ligands based on the steady-state behavior of RMSD of all target proteins in the presence of F1094-0201. Furthermore, our results suggested that the overall structure of the protein-ligand complex had not undergone any significant conformational changes.

#### 2.4.2. Root Mean Square Fluctuation (RMSF)

**Figure 6:**
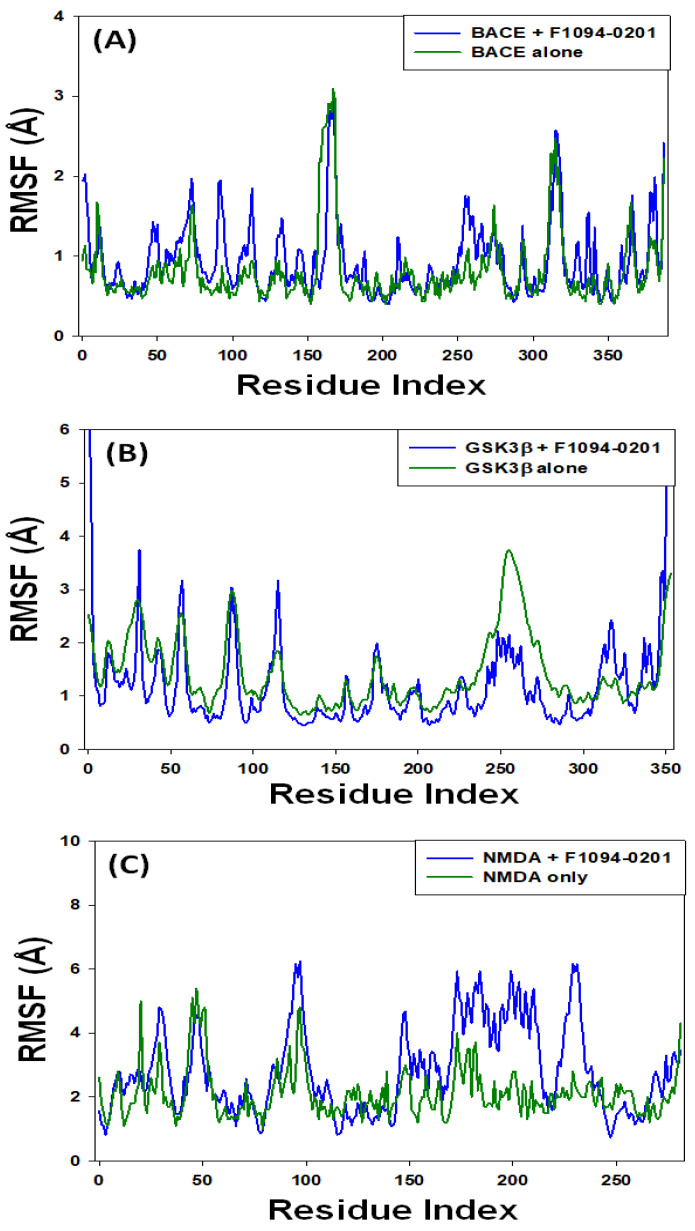
Variation in the root mean square fluctuation (RMSF) of (**A**) BACE, (**B**) GSK3β, and (**C**) NMDA, in the absence or presence of F1094-0201 as a function of simulation time.

The monitoring of RMSF is often used to identify the local conformational changes in a protein’s side chains caused by the binding of a ligand [73]. Here, the RMSFs of BACE, GSK3β and NMDA were accessed in the presence or absence of the ligand F1094-0201 (Figure 6). Fluctuations at proteins’ N- and C-terminals are due to their higher flexibilities. The results indicate that the RMSF plots of target proteins in the presence of F1094-0201 were almost overlapping with the RMSF plot of target proteins alone. The average RMSF values of BACE, GSK3β, and NMDA in the presence of F1094-0201 were 0.88 ± 0.03 Å, 0.94 ± 0.06 Å, and 0.79 ± 0.05 Å, respectively, while the average RMSF values of BACE, GSK3β, and NMDA in the presence of F1094-0201 were 0.94 ± 0.06 Å, 1.14 ± 0.08 Å, and 2.82 ± 0.09 Å, respectively. These findings indicate that the protein-ligand combination is stable in nature and that the binding of the F1094-0201 compound did not significantly alter the overall structure of the target proteins.

#### 2.4.3. The Radius of Gyration (Rg) and Solvent-Accessible Surface Area (SASA)

**Figure 7:**
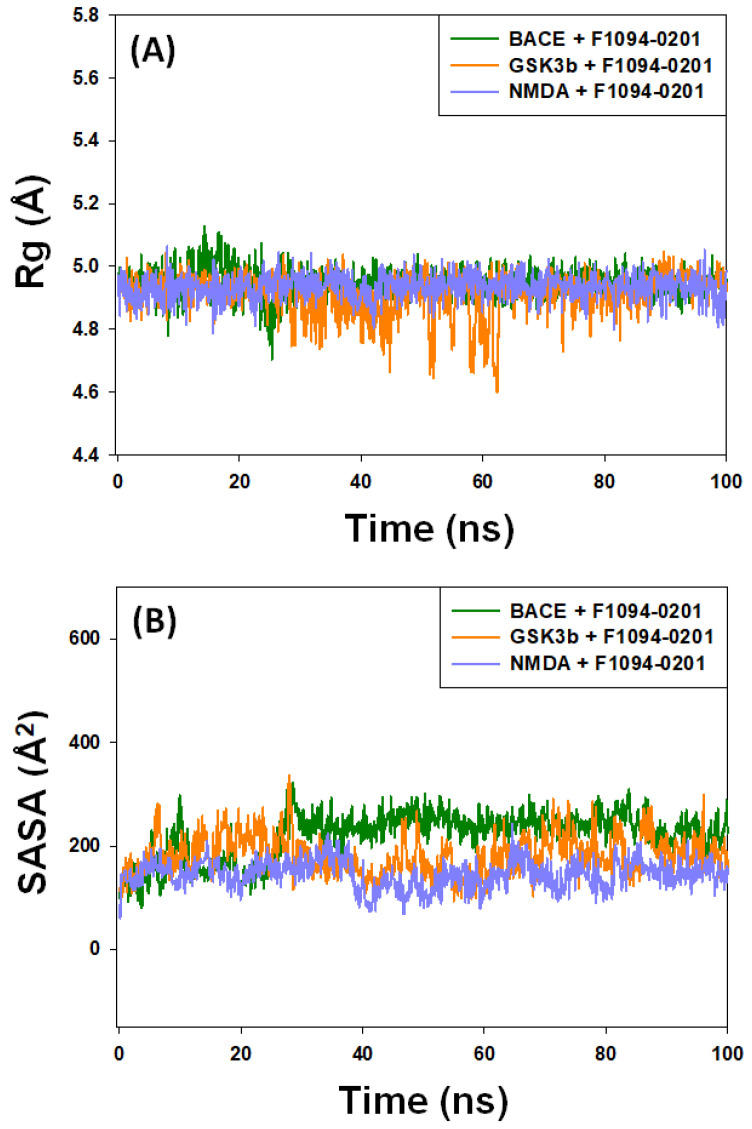
Dependency of (**A**) radius of gyration (Rg) and (**B**) solvent accessible surface area (SASA) of target proteins (BACE, GSK3β, and NMDA) in the presence of F1094-0201 on simulation time.

The radius of gyration measures the protein-ligand complex’s compactness, and the solvent-accessible surface area determines its exposure to solvent molecules. Each of these characteristics sheds light on the protein-ligand complex’s stability throughout the simulation [73]. In this study, the Rg of BACE, GSK3β, and NMDA was determined in the presence of F1094-0201 during 30–100 ns, as shown in Figure 7A. The Rg of BACE-F1094-0201, GSK3β-F1094-0201, and NMDA-F1094-0201 complexes varied in the range of 4.72–5.15 Å, 4.62–5.01 Å, and 4.79–5.06 Å, respectively, with an average value of 5.02 ± 0.07 Å, 4.88 ± 0.12 Å, and 4.94 ± 0.08 Å, respectively. Similarly, the SASA of BACE, GSK3β, and NMDA was determined in the presence of F1094-0201 during 30–100 ns (Figure 7B). The SASA of BACE-F1094-0201, GSK3β-F1094-0201, and NMDA-F1094-0201 complexes fluctuated in the range of 218.4–253.7 Å^2^, 104.2–271.8 Å^2^, and 93.7–202.6 Å^2^, respectively, with an average value of 272.5 ± 16.1 Å^2^, 201.7 ± 27.9 Å^2^, and 178.2 ± 20.4 Å^2^, respectively. These findings support the stability of protein-ligand complexes by showing that fluctuations in Rg and SASA of the protein of interest in the presence of F1094-0201 did not diverge significantly.

#### 2.4.4. Secondary Structure Elements (SSE)

**Figure 8:**
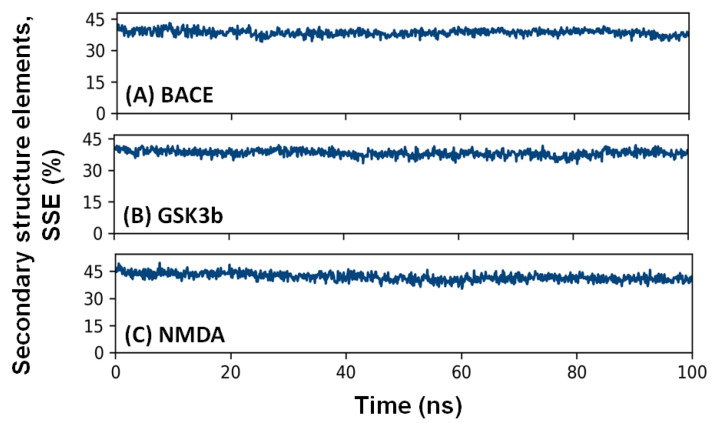
Change in the total secondary structure elements (SSE) of (**A**) BACE, (**B**) GSK3β, and (**C**) NMDA in the presence of F1094-0201, as a function of simulation time.

Monitoring the variations in a protein’s secondary structure elements (SSE) in a protein-ligand complex is significant for evaluating any structural changes in a protein due to ligand binding [74]. Here, we have evaluated the total SSE (α-helix + β-sheet) of BACE, GSK3β, and NMDA in the presence of F1094-0201 as a function of simulation time (Figure 8). The average SSE of BACE, GSK3β, and NMDA in complex with F1094-0201 was 38.56 ± 1.84% (α-helix = 5.57 ± 0.91% and β-sheet = 33.00 ± 2.22%), 38.11 ± 1.54% (α-helix = 22.09 ± 1.08% and β-sheet = 16.02 ± 1.87%), and 41.92 ± 2.56% (α-helix = 22.09 ± 1.37% and β-sheet = 19.84 ± 1.98%), respectively. The results indicate that the total SSE of all the targeted proteins in the presence of F1094-0201 did not undergo significant changes, thereby implying a stable protein conformation in the protein-ligand complex.

#### 2.4.5. Contacts between Protein and Ligand

**Figure 9:**
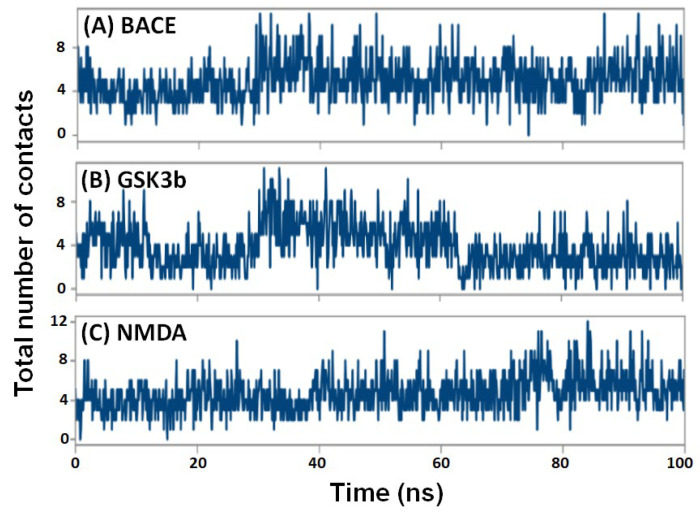
Change in the total number of contacts formed by F1094-0201 with (**A**) BACE, (**B**) GSK3β, and (**C**) NMDA as a function of simulation time.

The total number of contacts formed between protein and ligand was also evaluated over the simulation (Figure 9). The total number of interactions between ligand and protein in BACE-F1094-0201, GSK3β-F1094-0201, and NMDA-F1094-0201 complexes varied in the range of 0–11, 0–11, and 0–12, respectively. During the 30–100 ns simulation, the average contacts between F1094-0201 and BACE, GSK3β, and NMDA were 7, 5, and 6, respectively.

**Figure 10:**
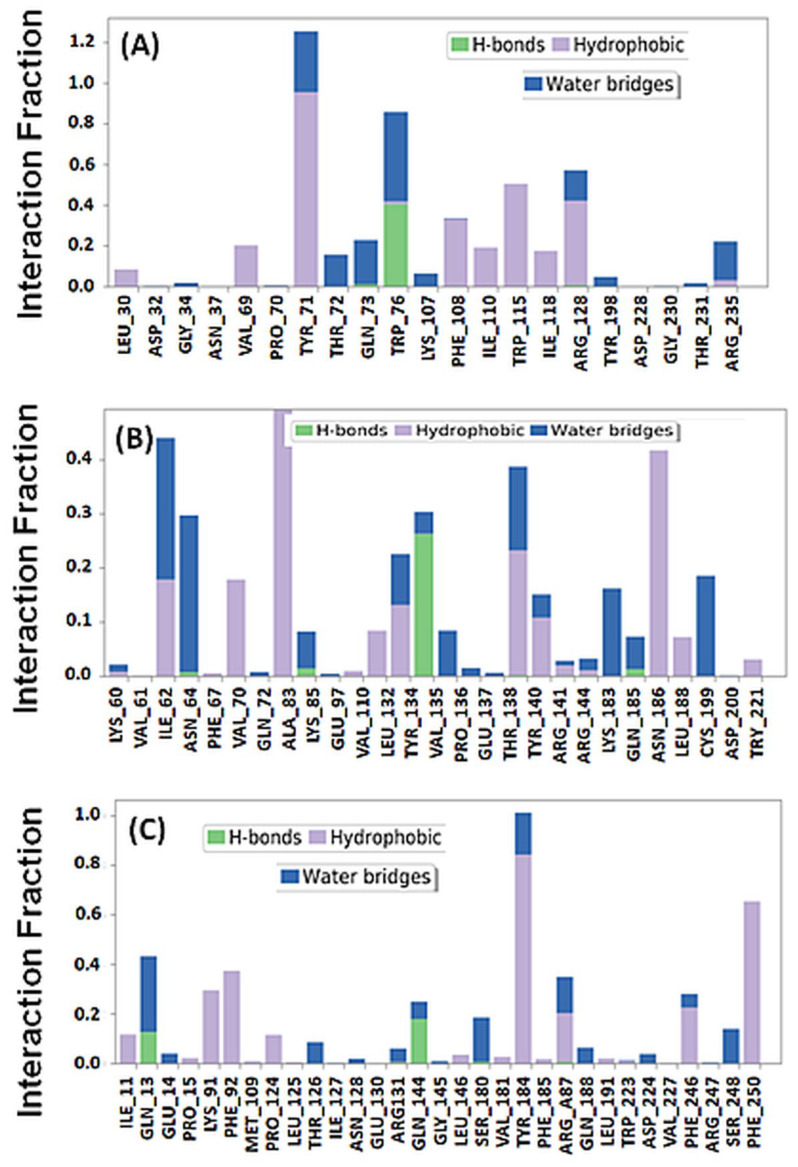
Interaction of F1094-0201 with (**A**) BACE, (**B**) GSK3β, and (**C**) NMDA, during simulation.

Further, the overall interaction pattern of F1094-0201 with BACE, GSK3β, and NDMA was also evaluated, as shown in Figure 10. BACE formed hydrophobic interactions with Tyr71, Lys107, Ile110, and Ile118, hydrogen bond with Trp76 for a significant simulation duration (Figure 10A). Similarly, GSK3β interacted with F1094-0201 through hydrophobic interactions with several catalytic amino acid residues (Ile62, Val70, Ala83, Leu132, Thr138, Tyr140, Gln185, and Leu188). F1094-0201 also formed a hydrogen bond with Tyr134 of GSK3β for a significant simulation duration (Figure 10B). Also, NMDA formed hydrophobic interactions with F1094-0201 through Phe16, Phe92, Tyr184, Phe185, Val227, and Ser248, and hydrogen bond with Arg131 (Figure 10C). These observations further emphasize that the F1094-0201 remained inside the binding pocket of target proteins and formed a stable protein-ligand complex.

### 2.5. Analysis of Prime/MM-GBSA Free Energy

Free energy calculation by Prime/MM-GBSA is a versatile and accurate method to evaluate the stability of a protein-ligand complex in the presence of solvent molecules [75]. Here, free energy and its constituents were determined using the Prime/MM-GBSA approach, and the results are presented in Table 3. Amongst the target proteins, F1094-0201 formed the most stable complex with BACE, followed by NMDA and GSK3β. The free energies of BACE-F1094-0201, GSK3β-F1094-0201, and NMDA-F1094-0201 complex formation were −73.78 ± 4.31 kcal mol^−1^, −72.77 ± 3.43 kcal mol^−1^, and −52.51 ± 2.85 kcal mol^−1^ respectively. In all the protein-ligand complexes, van der Waal forces, Coulombic forces, packing interactions, and lipophilic interactions favored the formation of a stable complex. Conversely, covalent interactions and solvation-free energy opposed the formation of a stable protein-ligand complex.

## 3. Materials and Methods

### 3.1. Computational Hardware and Software

Target proteins’ three-dimensional (3D) crystallographic structures have been retrieved from the PDB database at http://www.rcsb.org/pdb/ (accessed on 12 December 2020). Through Autodock vina-enabled PyRx software, molecular docking was carried out, and the Lamarckian genetic method was used as a scoring function [76,77]. The visualization of molecular interactions was analyzed through the Biovia Discovery Studio visualizer [78]. The molecular dynamics simulation study was performed by Desmond (Schrodinger-2020, LLC, New York, NY, USA) software. An Intel Xenon (E3-1245-8C) workstation powered by NVIDIA Quadro P5000 with a 3.50 GHz processor and 28 GB RAM was used for the computational study.

### 3.2. Preparation of Ligands and Proteins

The 2029 natural product-like compounds library was retrieved in .sdf format from Life Chemicals (www.lifechemicals.com) as accessed on 2 November 2020. After that, all the compounds were energy minimized using a universal force field and converted to Autodock suitable “.pdbqt” format. Moreover, for the 3D structure of target proteins, we used GSK3β co-crystallized with a substrate (Adenosine-5′-Diphosphate) with a resolution of 2.10 Å (PDB Id: 1J1C), the high-resolution (1.90 Å) co-crystal structures of the NR1 S1S2 ligand-binding core with the antagonist 5,7-dichloro kynurenic acid (DCKA) well-known as an NMDA-receptor (PDB Id: 1PBQ), and BACE enzyme co-crystallized with hydroxyethyl amine inhibitor at 2.55 Å resolution (PDB Id: 1W51) were downloaded from the PubChem database [59,65,69]. After that, all the heteroatoms, including native ligands and water molecules, were removed from target proteins and hydrogens (polar only) were added. Then, geometric optimization and energy minimization of these edited target proteins were performed using PyRx built-in tool. The finalized proteins were later converted to the “.pdbqt” format.

### 3.3. Molecular Docking

Virtual screening of natural product-like compounds against target proteins was performed through PyRx-Python 0.8, a freely available tool coupled with AutoDock 4.2, as described earlier [8]. The ligands were docked individually after protocol validation by docking native ligands in the active site coordinates of the co-crystallized structure. The grid box of size 60 × 60 × 60 Å for target proteins of active site coordinates were finalized through the discovery studio visualizer at the attributes of native ligands docked in their specific proteins. The grid box was centered at 21.61 × 17.28 × −8.57 Å for GSK3β, at 5.61 × 38 × −17.16 Å for NMDA-receptor, and at 73.79 × 54.27 × 11.51 Å for BACE-1, respectively. The exhaustiveness was set to 8, and all other docking parameters were set to default values. The binding energy (ΔG) of best poses was used to calculate the binding affinity (Kd) with the following equation [8]:

$$\Delta G=-RT\;lnK_{d}$$

In this equation, *R* stands for Boltzmann’s gas constant, and *T* stands for temperature.

### 3.4. Prediction of Drug-Likeness, Pharmacokinetics, and Physicochemical Properties

SwissADME (http://www.swissadme.ch) web-based tool was used and accessed on 15 February 2021, for predicting the properties of the 10 best docking hits such as molecular weight (MW), hydrogen bond donors (HBD), hydrogen bond acceptor (HBA), human gastrointestinal absorption (HIA), the permeability of blood-brain barrier (BBB), rotatable bond (RB), the fraction of sp3 (Fsp3) carbon atoms, cLogP value, and Lipinski’s rule of five violation [79].

### 3.5. Molecular Dynamics Simulation

As previously reported, Desmond (Schrodinger-2020, LLC, New York, NY, USA) was employed to run a molecular dynamics simulation to evaluate the stability of target proteins with the best-shortlisted molecule after molecular docking and ADME study [8,66]. Briefly, the simulation was performed inside an orthorhombic box after placing the docked protein-ligand complex at the center at a 10 Å distance from the boundaries. TIP3P water molecules were used to solvate the box, and the appropriate Na^+^ or Cl^−^ counterions were added to neutralize it. To simulate physiological circumstances, 150 mM of sodium chloride was added. The system’s energy was minimized through the OPLS3e forcefield by performing 2000 iterations with a convergence criterion of 1 kcal/mol/Å. Finally, a 100 ns production run was conducted at 298 K temperature and 1 bar pressure, which were kept constant using a Nose–Hoover Chain thermostat and a Martyna–Tobias–Klein barostat [80,81]. The energies and structures were recorded every 10 ps, with the time-step fixed at 2 fs. The molecular dynamics simulation results have been investigated for the root mean square deviation (RMSD), root mean square fluctuation (RMSF), the radius of gyration (Rg), solvent accessible surface area (SASA), secondary structure elements (SSE), and molecular interactions established between ligand and protein during simulation. The findings of each experiment were run independently, in triplicate, and are shown as mean ± standard errors.

### 3.6. Free Energy Calculations Using Prime/MM-GBSA

The free energy of interaction between protein and compound was calculated using Prime (Schrodinger-2020, LLC, New York, NY, USA) with the help of the MM-GBSA approach. Here, the free energies were calculated on the final 10 ns of the equilibrated trajectories. The docked protein-ligand complex was first optimized by molecular mechanics using Prime, followed by energy minimization by OPLS-AA forcefield using GBSA (Generalized Born Surface Area) continuum solvent model. Finally, the binding free energy of a protein-ligand complex was computed using the following equation:

$$r\;\Delta G_{Bind}=\Delta G_{Coulomb}+\Delta G_{vdW}+\Delta G_{Covalent}+\Delta G_{H-bond}+\Delta G_{Sol\_Lipo}+\;\Delta G_{Solv\_GB}+\Delta G_{Packing}+\Delta G_{Self-contact}$$

where Δ*G*_Bind_, Δ*G*_Coulomb_, Δ*G*_vdW_, Δ*G*_Covalent_, Δ*G*_H-bond_, Δ*G*_Sol_Lipo_, Δ*G*_Solv_GB_, Δ*G*_Packing_, and Δ*G*_Self-contact_ represent total free energy of protein-ligand complex, Coulombic free energy, van der Waals’ interactions free energy, covalent bonds free energy, hydrogen bonds free energy, the free energy of the surface area, Solvation free energy, packing free energy, and free energy of self-contact respectively.

## 4. Conclusions

Out of 2029 natural product-like compounds, F1094-0201 displayed the best drug-likeness, pharmacokinetics, and physiological properties that can cross the blood-brain barrier, and high GI absorption, based on the results of the virtual screening and molecular dynamics simulation (MDS) study. The MDS results indicated the establishment of a stable association between proteins and ligands based on the steady-state behavior of RMSD of all target proteins in the presence of F1094-0201. The RMSF plots of target proteins in the presence of F1094-0201 almost overlap with the RMSF plot of target proteins alone. Fluctuations in Rg and SASA of protein of interest in the presence of F1094-0201 did not diverge significantly. The total SSE of all the target proteins in the presence of F1094-0201 did not undergo any significant changes. These observations further emphasize that the F1094-0201 remained inside the binding pocket of target proteins and formed a stable protein-ligand complex. The free energies (MM/GBSA) results indicated that in all the protein-ligand complexes, van der Waal forces, Coulombic forces, packing interactions, and lipophilic interactions favored the formation of a stable complex. Amongst the target proteins, F1094-0201 formed the most stable complex with BACE, followed by NMDA and GSK3β. These attributes of F1094-0201 indicate it as a possible option for the management of pathophysiological pathways associated with AD. Our perspective is to utilize the beneficial effects of the lead compound (F1094-0201) against neurological disorders, and further, our strategy is to analyze the therapeutic potency of this compound by in-vivo experiments; thereafter, the drug candidate can be evaluated through clinical trials and get approval for patient care. In this study, the candidate drug’s neuroprotective potentials were solely examined using in-silico methods, thereby opening the door for verification of its clinical effectiveness using in-vitro and in-vivo biological systems.

## Figures and Tables

**Table 1:** Binding energy and binding affinity data through molecular docking study for the best 10 hits against BACE, GSK3β, and NMDA.

		Binding Energy (ΔG) kcal mol^−1^			Binding Affinity (*K*_d_) M^−1^		
	Targets	BACE (1w51)	GSK3β (1j1c)	NMDAr (1pbq)	BACE (1w51)	GSK3β (1j1c)	NMDAr (1pbq)
	ID number						
C1	F0870-0001	−12.0	−11.2	−12.3	6.25 × 10^8^	1.62 × 10^8^	1.04 × 10^9^
C2	F1094-0201	−12.0	−10.6	−11.7	6.25 × 10^8^	5.89 × 10^7^	3.77 × 10^8^
C3	F0882-0397	−11.0	−10.4	−12.3	1.16 × 10^8^	4.20 × 10^7^	1.04 × 10^9^
C4	F1217-0041	−11.0	−9.8	−11.6	1.16 × 10^8^	1.53 × 10^7^	3.18 × 10^8^
C5	F1094-0199	−11.1	−9.7	−10.9	1.37 × 10^8^	1.29 × 10^7^	9.77 × 10^7^
C6	F1094-0205	−11.2	−9.9	−10.9	1.62 × 10^8^	1.81 × 10^7^	9.77 × 10^7^
C7	F1094-0196	−11.1	−9.9	−10.8	1.37 × 10^8^	1.81 × 10^7^	8.25 × 10^7^
C8	F1094-0198	−11.4	−9.8	−10.5	2.27 × 10^8^	1.53 × 10^7^	4.97 × 10^7^
C9	F1094-0206	−10.9	−9.9	−10.5	9.77 × 10^7^	1.81 × 10^7^	4.97 × 10^7^
C10	F3161-0307	−11.5	−9.3	−10.1	2.69 × 10^8^	6.56 × 10^6^	2.53 × 10^7^
RL1	Non-peptidic inhibitor	−10.6	ND	ND	5.89 × 10^7^	ND	ND
RL2	Adenosine-5′-Diphosphate	ND	−7.7	ND	ND	4.41 × 10^5^	ND
RL3	5,7-Dichlorokynurenic acid	ND	ND	−7.2	ND	ND	1.90 × 10^5^

**Table 2:** Physicochemical, pharmacokinetics and drug-likeness properties of natural product-like compounds.

	Physicochemical Properties								Pharmacokinetics			Drug-Likeness	
	Formula	MW	RB	HBA	HBD	MR	TPSA	XLOGP3	GIA	BBB+	Pgp-S	Fcsp3	LV
C1	C_24_H_15_NO_6_	413.38	3	7	2	114.31	113.77	2.12	High	No	Yes	0.04	0
C2	C_26_H_19_NO_4_	409.43	2	5	0	119.78	59.75	5.18	High	Yes	No	0.15	0
C3	C_24_H_13_NO_5_	395.36	3	5	1	110.01	93.45	4.09	High	No	No	0	0
C4	C_26_H_19_NO_4_	409.43	3	5	0	119.78	59.75	5.24	High	Yes	Yes	0.15	0
C5	C_22_H_17_NO_6_S	423.44	1	7	0	111.75	102.27	2.85	High	No	No	0.27	0
C6	C_26_H_23_NO_4_	413.47	3	5	0	121.54	59.75	5.33	High	Yes	Yes	0.31	0
C7	C_24_H_21_NO_4_	387.43	1	5	0	112.4	59.75	5.09	High	Yes	Yes	0.33	0
C8	C_23_H_19_NO_4_	373.4	1	5	0	107.6	59.75	4.55	High	Yes	No	0.300	0
C9	C_28_H_23_NO_6_	469.49	5	7	0	132.76	78.21	5.18	High	No	Yes	0.21	0
C10	C_26_H_32_Cl_2_O_2_	447.44	1	2	1	125.46	37.3	7.19	Low	No	No	0.65	1

Molecular weight: MW, #Rotatable bonds: RB, #H-bond acceptors: HBA, #H-bond donors: HBD, Molar refractivity: MR, topological polar surface area: TPSA, cLogP value: XLOGP3, gastrointestinal absorption: GIA, blood-brain barrier permeation: BBB+, Pgp substrate: Pgp-s, Fraction Csp3: Fcsp3, Lipinski #violations: LV.

**Table 3:** Components of free energy of target protein with the identified inhibitor using Prime/MM-GBSA approach.

Target Proteins	ΔE_MM_			ΔG_Solv_ or ΔG_SolGB_	ΔG_Self contact_	ΔG_H-bond_	ΔG_SA_ or ΔG_Sol_Lipo_	ΔG_Packing_	ΔG or ΔG_Bind_
	ΔG_Coulomb_	ΔG_vdW_	ΔG_Covalent_						
BACE	−8.66 ± 0.73	−48.86 ± 3.13	0.70 ± 0.06	15.30 ± 0.81	0	−0.26 ± 0.03	−26.48 ± 1.97	−5.52 ± 0.45	−73.78 ± 4.31
GSK3β	−4.39 ± 0.56	−36.71 ± 2.42	0.71 ± 0.03	18.78 ± 1.04	0	−0.14 ± 0.02	−26.45 ± 2.02	−4.32 ± 0.32	−52.51 ± 2.85
NMDA	−5.04 ± 0.91	−44.92 ± 3.48	1.34 ± 0.10	11.67 ± 0.87	0	−0.02 ± 0.02	−31.23 ± 2.29	−4.57 ± 0.28	−72.77 ± 3.43

Note: All the energies are represented in kcal/mol. Minimized molecular mechanics energy (ΔE_MM_), coulomb energy (ΔG_Coulomb_), van der Waals’ energy (ΔG_vdW_), covalent binding energy (ΔG_Covalent_), solvation energy (ΔG_Solv_ or ΔG_SolGB_), energy due to self-contact (ΔG_Self-contact_), energy due to H-bonds (ΔG_H-bond_), lipophilic energy (ΔG_SA_ or ΔG_Sol-Lipo_), and binding energy (ΔG or ΔG_Bind_).

## Data Availability

Not applicable.
